# Oral tryptophan supplementation to suckling piglets affecting hypothalamic serotonin metabolism, behavior, and growth in the nursery period under social mixing stress

**DOI:** 10.1093/jas/skaf299

**Published:** 2025-08-30

**Authors:** Tiago J Pasquetti, Alexa R Gormley, Jiyao Guo, Paulo C Pozza, Sung Woo Kim

**Affiliations:** Department of Animal Science, North Carolina State University, Raleigh, NC, USA; Department of Animal Science, North Carolina State University, Raleigh, NC, USA; Department of Animal Science, North Carolina State University, Raleigh, NC, USA; Departamento de Zootecnia, Universidade Estadual de Maringá, Maringá, Brazil; Department of Animal Science, North Carolina State University, Raleigh, NC, USA

**Keywords:** feed intake, mixing stress, pigs, serotonin, tryptophan

## Abstract

Tryptophan (**Trp**) is the rate-limiting substrate for the synthesis of serotonin (5-hydroxytryptamine; **5-HT**) in the brain, which plays a central role in the regulation of stress sensitivity and feed intake. The objective of this study was to evaluate the effect of oral Trp supplementation to suckling piglets on 5-HT metabolism, growth performance, and behavior during the suckling (14 to 21 d of age) and nursery (21 to 56 d of age) periods, under social mixing stress at weaning. Eighty 14-d-old suckling piglets from 10 sows (8 piglets per litter) were allotted to 2 treatments. The 2 treatments (0.389 g alanine + 0.430 g glucose/kg body weight (**BW**)/d or 0.467 g Trp/kg BW/d) were given orally to piglets 7 times daily, beginning at 0700 h in 2 h intervals, from day 14 to day 21 of age (suckling period). Alanine (Ala) was used to balance the nitrogen content when Trp was added. Growth performance was measured from day 14 to day 21 of age (suckling period) and from day 21 to day 56 of age (nursery period) in 2 phases. On day 21, 12 piglets from 12 L (6 pigs/treatment) were selected and euthanized to collect hypothalamus to measure 5-HT and 5-hydroxyindoleacetic acid (**5-HIAA**) concentrations. The remaining piglets were weaned and moved to nursery pens. Pigs within the same treatment and sex, but from different litters, were paired and housed together (2 pigs/pen). All pigs were fed the same diet during the nursery period. Normal and aggressive behaviors were recorded for 24 h immediately after weaning. Oral Trp supplementation during the suckling period increased (*P* < 0.05) hypothalamic concentration of 5-HT and 5-HIAA at weaning, whereas growth performance was not affected. However, oral Trp supplementation tended to increase (*P* = 0.057) the frequency of visits to the feeders during social mixing stress. In conclusion, oral Trp supplementation during the suckling period increased hypothalamic serotonin and could influence eating behavior postweaning.

## Teaser

Tryptophan is a rate-limiting substrate in the synthesis of serotonin, which has roles in managing the stress response and feeding patterns in animals. The supplementation of oral tryptophan to suckling piglets increased serotonin and related metabolites in pigs under social mixing stress postweaning.

## Introduction

The postweaning period is a critical determinant for the future productivity of pigs as the nutritional, physiological, and behavioral stress imposed at weaning can negatively influence overall health status, thus affecting lifetime performance (Collins et al., 2017; Kim and Duarte, 2021; Zheng et al., 2021). Upon weaning, pigs are transported to a new location and placed into pens grouped based on common parameters such as body weight (**BW**), sex, and health status (Kim and Hansen, 2022). As pigs are mixed with unfamiliar pigs, they will establish a social hierarchy, which triggers aggressive behaviors for several days after the initial co-mingling (Jensen, 1994). This period of intense social stress can impair performance, welfare, and health (Ruis et al., 2000; Shen et al., 2012a; Zhao et al., 2013).

Cerebral serotonin, 5-hydroxytrptamine (**5-HT**), metabolism affects behavioral and emotional responses of animals (Gutknecht et al., 2015). In the brain, the availability of the amino acid tryptophan (**Trp**), as a substrate, serves as the rate-limiting step in the synthesis of 5-HT. Therefore, it is thought that the availability of Trp could influence the production of cerebral 5-HT, thereby influencing the behavioral and stress responses. As such, it has been shown that dietary Trp modulates aggressive behavior in different animal species (Gibbons et al., 1979; Shea et al., 1990; Shen et al., 2012a). In pigs, it was found that dietary Trp supplementation at 0.6% of the diet increased feed efficiency and the hypothalamic concentration of 5-HT (Shen et al., 2012a) and the benefits of Trp exerted on the performance and stress physiology of pigs under social mixing stress were maximized when the daily total Trp intake was 10.8 g (Shen et al., 2012b). Additionally, studies have shown that dietary Trp supplementation can improve intestinal barrier function and enhance innate immune responses under challenge conditions (Koopmans et al., 2006; Liu et al., 2019), which suggests that Trp may provide further benefits, in addition to serving as a precursor for 5-HT production in pigs.

As such, it was hypothesized that providing supplemental Trp to suckling piglets could help them to better adapt to challenges faced at weaning, particularly social mixing stress, thus enhancing growth performance in the nursery period. The objective of this study was to evaluate the effect of oral Trp supplementation to suckling piglets on 5-HT metabolism, growth performance, and behavior during the suckling period (from day 14 to day 21 of age) and subsequent nursery period (from day 22 to day 56 of age) under social mixing stress after weaning.

## Materials and Methods

The animal experimental protocol used in this study (13-118-A) was approved by the North Carolina State University Animal Care and Use Committee.

### Animals and experimental design

On day 14 of age, 10 L with a minimum of 4 barrows and 4 gilts were selected. All piglets from 10 litters with an initial BW of 4.62 ± 0.09 kg were assigned to 2 treatments based on a randomized complete block design with sex serving as the block. The 2 treatments were 1) CON: oral supplementation of Ala and glucose (0.38 and 0.42 g/kg BW/d, respectively), and 2) TRP: oral supplementation of Trp (0.46 g/kg BW/d). Alanine and glucose were used to balance nitrogen (N) and metabolizable energy (**ME**) between the 2 treatments and Ala was chosen because previous studies showed that Ala is extensively catabolized and does not exert toxic or additional biological functions (Kim et al., 2004; Mateo et al., 2007). Piglets received their respective treatments 7 times daily (from 0700 to 1900 h with 2 h intervals) from day 14 to day 21 of age. At day 21 of age, piglets were weaned, weighed, and moved to nursery pens (1.73 × 0.83 m; 1.44 m^2^) with metal screen floors, a nipple drinker, and a 2-hole self-feeder. Pigs within a treatment and sex, but from different litters, were paired and housed together (2 pigs/pen) using a randomized complete block design. All weaned pigs were fed the same diet during the nursery period, from days 21 to 56 of age.

### Dietary treatments and handling of suckling piglets

According to the *Nutrient Requirements of Swine* (NRC, 2012), the daily requirement of Trp for pigs weighing 5 to 7 kg BW is 0.7 g/pig/d, which is approximately 0.117 g/kg BW/d. Based on the results from Shen et al. (2012a, 2012b), the amount of Trp utilized for the treatment group was set to be 4-fold above the requirement (0.117 g × 4 = 0.468 g/kg BW/d). Based on the BW of piglets, the amount of daily Trp supplementation was calculated (Table 1).

**Table 1.** Nutrient composition of dietary treatments offered daily to suckling piglets

	Treatment	
Item	CON	TRP
Tryptophan, g/kg BW^1^	0.000	0.467
Alanine, g/kg BW^2^	0.389	0.000
Glucose, g/kg BW^3^	0.430	0.000
Crude protein, g/kg BW	0.400	0.400
ME, kcal/kg BW	2.890	2.890

^1^L-Tryptophan: 82.45% of CP and 6,180 kcal/kg of ME.

^2^Alanine: 102.45% of CP and 3,725 kcal/kg of ME.

^3^Glucose: 3,334 kcal/kg of ME.

Based on the amount of Trp to be supplemented (per kg BW/d), the daily amount of crude protein (0.467 g Trp × 0.855 g/100 g of CP = 0.40 g of CP/kg BW/d) and ME (0.467 g Trp × 6.18 kcal/g ME = 2.89 kcal ME/kg BW/d) contributed from Trp were calculated. For the control group, Ala and glucose were used to provide equal amount of CP (0.389 g Ala × 0.1025 g/100 g of CP = 0.40 g of CP/d/kg BW) and ME (0.389 g Ala × 3.73 kcal/g = 1.45 kcal ME/d/kg BW); then, glucose was used to provide an equal amount of ME (0.430 g glucose × 3.33 kcal/g ME = 1.44 kcal/d/kg BW), totaling 2.89 kcal of ME/kg BW/d.

Treatments were provided to suckling piglets individually in a solution to facilitate oral supplementation. Predetermined amounts of Trp or Ala + glucose were dissolved in distilled water (2.7 mL/kg BW), and the treatment solutions were prepared daily. A syringe (3 mL) was used to provide treatment solutions to piglets, which were given 7 times daily from 0700 h to 1900 h in 2 h intervals from day 14 to day 21 of age prior to weaning. Each oral supplementation took less than 5 s. On day 14 and day 21 of age, piglets were weaned and weighed to calculate the average daily gain (**ADG**) during the suckling period.

### Hypothalamus and assays

On day 21 of age, after the last oral supplementation (1900 h), 6 litters from each treatment were randomly selected and 1 piglet per litter (12 piglets total; 6 per treatment; 3 gilts and 3 barrows) representing the median BW of each litter was selected and euthanized to collect the hypothalamus. Following Shen et al. (2012a), the brain was quickly removed, and the hypothalamus was dissected, snap frozen in liquid nitrogen, and then subsequently stored at -80 °C.

The hypothalamus was cut to yield a section weighing 0.5 g and the tissue was then homogenized (Tissuemiser, Thermo Fisher Scientific Inc., Rockford, IL) in a 1 mL buffer containing 28 mM of Tris-HCL, pH 7.4, 1mM of EDTA and 1mM of ethylene glycol tetra acetic acid. All procedures were conducted on ice. Homogenized hypothalamus was then centrifuged at 15,000 × *g* at 4 °C for 30 min. Supernatants were used to determine the concentrations of protein, 5-HT, and 5-hydroxyindoleacetic acid (5-HIAA).

Protein concentration was measured using a commercial kit (Thermo Fisher Scientific Inc.) as previously described by Shen et al. (2012b). Briefly, bovine serum albumin standards were prepared in 0.1 M perchloric acid. The standard and samples (10 μL) were added to 200μL of the working reagent, bicinchoninic acid, and prepared in duplicates according to the manufacturer’s recommendations. Concentrations of 5-HT and 5-HIAA in the hypothalamus were measured using commercial ELISA kits (IBL, Hamburg, Germany) as described by Shen et al. (2012b). Samples were tested in duplicate for 5-HT and triplicate for 5-HIAA. Concentrations of 5-HT and 5-HIAA were expressed in ng/mg of hypothalamic protein. Intra-assay CV for 5-HT and 5-HIAA were 9.2% and 12.1%, respectively.

### Growth performance and behavior of nursery pigs

Piglets were weaned on day 21 of age and moved to nursery pens, in which 2 pigs within the same treatment and sex, but from different litters of origin, were housed together to introduce social stress. All pigs consumed a common diet formulated to meet the requirements by the NRC (2012) in 2 phases.

To evaluate the effects of dietary Trp supplementation during the suckling period on pig behavior at weaning, the behavior of each pig was recorded in real time with a frame rate of 12 frames/s, captured using a ceiling-mounted circuit-closed television camera (Q-See QD28414C4, Digital Peripheral Solutions Inc., Anaheim, CA), connected to a digital video recorder (Q-See QSDT8DP, Digital Peripheral Solutions Inc.). Cameras were prepared and the system was turned on before the pigs were moved to nursery pens.

Behavior was classified as either normal, with normal behaviors being lying, standing, sitting, eating, and drinking, or aggressive, with aggressive behaviors being fighting and aggression. Aggressive behaviors were considered part of the standing behavior and are defined as agonistic interactions between pigs. Fighting was a mutual behavior between two pigs and included parallel pressing, inverse parallel pressing, and mutual bite, as previously described by Shen et al. (2012b). Aggression was 1-sided antagonistic interactions between pigs, including biting, knocking, and levering (Rushen, 1987; D’Eath, 2002).

Once the pigs were mixed, behavior was recorded continuously for 24 h and was measured as previously described (D’Eath, 2002; Mateo et al., 2006; Shen et al., 2012b). The instantaneous scan-sampling method was used to determine the percentage of time spent on specific behaviors, normal, or aggressive, during the 24 h observation period. During this 24 h period, each pig was observed every 2 min, a total of 720 observations per pig for the entire observation period. To minimize variation in the collected data, all behaviors were analyzed by the same observer, who was trained prior to the start of behavior data analysis. The number of observations and the total duration of fighting (parallel pressing, inverse parallel pressing, and mutual bite) and aggressive (biting, hitting, and levering movement) behaviors were evaluated as described by D’Eath (2002) and Li et al. (2006).

The aggressive behavior assessments were divided into 3 periods during the 24 h observation period. During the first period (from 1000 h to 1900 h), behavior was continuously observed after placing pigs together in the nursery room. During the second period (from 1900 h to 0400 h), behavior was observed from 1900 h to 2000 h, 2300 h to 2400 h, and 0300 h to 0400 h (for 1 h within 4 h intervals) due to lack of activity as most pigs were sleeping. During the third period (from 0400 h to 1000 h), behavior was continuously observed, as in the first period. Pigs remained in the same pen in which they were initially placed until day 56 of age, or week 5 postweaning. Pigs and feeders, including remaining feed, were weighed weekly to calculate ADG, average daily feed intake (**ADFI**), and gain to feed ratio (**G:F**).

### Statistical analyses

Data for each response were analyzed using the MIXED Procedure of SAS (SAS Inc., Cary, NC) based on a randomized complete block design with treatment and sex as fixed effects. Before weaning, each individual piglet was considered an experimental unit, whereas the pen was considered the experimental unit for performance and behavior after weaning because two pigs in the same pen shared feed in one feeder. Normal behavior data were recorded as a count (nº) and then expressed as a proportion (%) of total normal behaviors expressed. Aggressive and fighting behavior data were recorded as a frequency (nº) or duration (s). All recorded behavior data were transformed into logarithms (Log10) before statistical analysis, considering that the data were normally distributed. Statistical differences were considered significant when *P *< 0.05 and were considered a tendency when 0.05 ≤ *P *< 0.10.

## Results

### Growth performance

The average initial BW of piglets day 14 of age was 4.62 ± 0.09 kg. During the suckling period, no differences in BW or ADG were observed (Table 2). In week 1 postweaning, no differences in ADG or ADFI were observed, however, G:F tended to increase (*P *= 0.094; 16.8%) by oral Trp supplementation given during suckling period. In week 2 postweaning, there again were no differences in ADG or ADFI, however, G:F was decreased (*P *< 0.05; 7.4%) by oral Trp supplementation given during the suckling period. In week 3 postweaning, no differences in ADG, ADFI, or G:F were observed. In week 4 postweaning, ADG was increased (*P *< 0.05; 10.2%) by Trp supplementation; however, ADFI was not affected by the treatments. As a result, G:F also was increased (*P *< 0.05; 5.6%) by Trp supplementation, in week 4 postweaning.

**Table 2.** Growth performance of pigs fed diets with or without additional Trp supplementation, through the suckling and nursery periods^1^

	Treatment^2^		SEM^3^	*P* value
Item	CON	TRP		
BW, kg				
Day 14 of age	4.6	4.6	0.1	0.878
Day 22 of age	6.3	6.4	0.5	0.480
Day 28 of age	7.2	7.4	0.2	0.214
Day 35 of age	9.4	9.4	0.3	0.893
Day 42 of age	12.3	12.3	0.3	0.881
Day 49 of age	15.9	16.4	0.4	0.309
Day 56 of age	20.7	21.3	0.4	0.207
ADG, g/d				
Day 14 to day 21 of age	287	296	0.01	0.467
Week 1 postweaning	69	83	0.01	0.133
Week 2 postweaning	320	295	0.02	0.257
Week 3 postweaning	413	414	0.02	0.962
Week 4 postweaning	519	572	0.03	0.040
Week 5 postweaning	681	700	0.03	0.467
Days 14 to 56 of age (experimental period)	395	408	0.01	0.176
ADFI, g/d				
Week 1 postweaning	152	162	0.01	0.294
Week 2 postweaning	400	401	0.02	0.973
Week 3 postweaning	722	726	0.02	0.870
Week 4 postweaning	927	965	0.04	0.301
Week 5 postweaning	1,219	1,262	0.03	0.188
Days 21 to 56 of age (nursery period)	675	696	0.02	0.237
G:F				
Week 1 postweaning	0.44	0.51	0.25	0.094
Week 2 postweaning	0.80	0.74	0.03	0.030
Week 3 postweaning	0.57	0.57	0.06	0.934
Week 4 postweaning	0.56	0.59	0.05	0.040
Week 5 postweaning	0.56	0.55	0.05	0.728
Days 21 to 56 of age (nursery period)	0.59	0.59	0.02	0.797

^1^ *n* = 68.

^2^CON: no Trp supplementation (0.380 g Ala + 0.430 g glucose/kg BW/d) fed from day 14 to day 21 of age; TRP: Trp supplementation (0.467 g Trp/kg BW/d) fed from day 14 to day 21 of age.

^3^Standard error of the mean.

### Hypothalamic serotonin and 5-HIAA

The concentration of 5-HT in the hypothalamus was 2.8 and 5.9 ng/mg of protein in the CON and TRP diets, respectively. Furthermore, the concentration of 5-HIAA in the hypothalamus was 59.1 and 104.6 µg/mg of protein in the CON and TRP diets, respectively. The 5-HIAA:5-HT were 22,216 and 20,148 in the CON and TRP diets, respectively. The concentration of 5-HT in the hypothalamus was increased (*P *< 0.05; 211%) by Trp supplementation, whereas the concentration of 5-HIAA in the hypothalamus had a tendency to be increased (*P *= 0.05; 177%) by Trp supplementation (Table 3). The 5-HIAA:5-HT did not differ between treatments.

**Table 3.** Effect of oral Trp supplementation on the concentration of hypothalamic serotonin (5-HT) and 5-hydoxyindolacetic acid (5-HIAA) in piglets on day 20 of age^1^

	Treatment		SEM^2^	*P-*value
Item	CON	TRP		
5-HT, ng/mg protein	2.8	5.9	0.9	0.030
5-HIAA, µg/mg protein	59.1	104.6	15.0	0.050
5-HIAA:5-HT	22,216	20,148	44	0.720

^1^ *n* = 6.

^2^Standard error of the mean.

### Normal and aggressive behavior

Regarding the effect of oral Trp supplementation on the normal behavior of pigs in the 24 h postweaning, pigs not supplemented with oral Trp during the suckling period exhibited the behaviors of lying (69.3%), sitting (1.5%), standing (28.4%), eating (0.4%), and drinking (0.4%) (Table 4). Pigs supplemented with oral Trp during the suckling period exhibited the behaviors of lying (70.1%), sitting (1.1%), standing (27.4%), eating (0.8%), and drinking (0.6%). Supplementation with Trp tended to increase (*P *= 0.057) the percentage of time spent displaying eating behaviors in pigs (Table 4). All other normal behavioral parameters were unaffected by the treatments.

**Table 4.** Effect of oral Trp supplementation on normal behavior of pigs in 24 h immediately post-weaning^1^^,^^2^

	Treatment		SEM^3^	*P*-value
Item	CON	TRP		
Lying, %	69.3	70.1	2.2	0.720
Sitting, %	1.5	1.1	0.2	0.120
Standing, %	28.4	27.4	2.1	0.660
Eating, %	0.4	0.8	0.2	0.057
Drinking, %	0.4	0.6	0.1	0.180

^1^ *n* = 17.

^2^Behaviors (lying sitting, standing, eating, and drinking) were measured for 24 h, and behaviors were recorded every two minutes, expressed as a percentage of total recorded behaviors.

^3^Standard error of the mean.

Regarding the effect of oral Trp supplementation on aggressive behavior of pigs in the 24 h postweaning, in the overall observation period, pigs exhibited fighting behaviors for 13.2 and 9.4 total observations in the CON and TRP treatments, respectively (Table 5). In the overall observation period, the pigs exhibited aggression for 631.6 and 661.1 total observations in the CON and TRP treatments. The aggressive behaviors, classified as fighting or aggression, of pigs were not affected by oral Trp supplementation in any of the observational periods (Table 5).

**Table 5.** Effect of oral Trp supplementation on aggressive behavior in the first 24 h immediately after weaning^1^

	Treatment			
Item	CON	TRP	SEM^2^	*P*-value
1000 h to 1900 h^3^				
Fighting^4^				
Frequency, nº	9.3	8.1	3.4	0.730
Total duration, s	287.2	307.2	96.7	0.880
Average duration, s	67.5	74.8	19.1	0.780
Aggression^5^				
Frequency, nº	368.0	434.0	93.3	0.450
1900 h to 0400 h^6^				
Fighting				
Frequency, nº	1.5	0.6	0.7	0.260
Total duration, s	49.6	12.1	28.3	0.310
Average duration, s	15.1	12.9	6.2	0.240
Aggression				
Frequency, nº	110.4	85.3	37.9	0.610
0400 h to 1000 h^7^				
Fighting				
Frequency, nº	2.5	0.8	1.3	0.150
Total duration, s	95.3	37.1	47.6	0.340
Average duration, s	32.8	29.3	19.7	0.890
Aggression				
Frequency, nº	165.7	148.1	53.8	0.800
Total period^8^				
Fighting				
Frequency, nº	13.2	9.4	5.1	0.420
Total duration, s	434.4	359.5	131.4	0.680
Average duration, s	76.30	74.2	19.8	0.940
Aggression				
Frequency, nº	631.6	661.1	151.5	0.800

^1^ *n* = 34.

^2^Standard error of the mean.

^3^Behavior was continuously monitored during this period.

^4^Frequency, total duration, and average duration of observed fighting were recorded; fighting was considered to be pressing, inverse parallel pressing, or mutual biting that occurred during each evaluation period.

^5^Frequency of observed aggressive behavior was recorded. Aggression was considered to be biting, hitting, or levering movements.

^6^Behavior was continuously monitored for 1 h in 4 h intervals, considering that most pigs were sleeping, during this time period.

^7^Behavior was continuously monitored during this period.

^8^Total period includes all measured data across the 24 h observational period.

## Discussion

It is well established that the stressors brought on at weaning, including separation from the sow, mixing with unfamiliar pigs, changes in ambient temperature, and dietary transitions can affect the growth performance of pigs, especially in the first few days after weaning (Campbell et al., 2013; Kim and Duarte, 2021). This period of intense stress can inhibit maturity of the gastrointestinal tract and immune function, which may leave pigs susceptible to diseases and decrease overall productivity (Zheng et al., 2021). Based on results from previously conducted studies (Shen et al., 2012a, 2012b), it was thought that supplementation of Trp in the suckling period could reduce stress at weaning caused by social mixing stress, thereby exerting positive effects on growth performance and stress behaviors in the subsequent nursery period.

Since 2012, the commercial production of Trp has increased by more than 50,000 tons of Trp produced annually, due to improvements in the industrial production of Trp, including optimizing bacteria-based fermentation strategies (Yin et al., 2024). Therefore, the inclusion of Trp in the diets of pigs to reduce stress at weaning could be economically feasible, given these recent improvements in production efficiency (Kim et al., 2024). Among the 20 amino acids that constitute proteins, Trp is found in relatively lesser amounts in the body and in the plasma compared to other amino acids (Kim et al., 2001; Le Floc’h et al., 2011). Production of 5-HT is one of the main physiological functions performed by Trp, although according to Wolf (1974), less than 1% of ingested Trp is used for its production. The metabolic pathway of kynurenine production by Trp is heavily favored over the production of 5-HT by Trp (Le Floc’h and Seve, 2007). Despite this, because 5-HT is related to the appetite regulation, sleep, impulsivity, aggression, sexual behavior, overall maintenance of the stress response, and can serve as a predictor of behavioral responses to social mixing stress (Soubrié, 1986; Clouard et al., 2023), several studies have directed their attention to the possible effects of dietary Trp on the production of 5-HT, and, therefore, its effects on the performance and behavior of pigs.

The rate of 5-HT synthesis, turnover, or activity of serotonergic system is reflected in the release of 5-HT, which can be quantified by the concentration of 5-HIAA or 5-HIAA:5-HT, where changes in Trp availability may affect serotonergic function (Höglund et al., 2019). In the present study, oral supplementation of Trp increased concentrations of 5-HT and 5-HIAA in the hypothalamus of piglets on day 20 of age. It is important to note that in the similar studies conducted by Shen et al. (2012a, 2012b) and Koopmans et al. (2006), the hypothalamus was collected after mixing of the animals, meaning the hypothalamic tissue was collected after pigs were subjected to social stress. In this study, to investigate the production of 5-HT in the hypothalamus prior to social mixing stress, the hypothalamus was collected after the last Trp supplementation, prior to weaning and the introduction to unfamiliar pigs. Further research could be performed to measure the concentration of 5-HT and 5-HIAA levels before and after stress events, which could more clearly display the turnover capacity of the serotonergic nervous system of animals and the capacity of 5-HT utilization during periods of stress.

The measured 5-HT and 5-HIAA concentrations in hypothalamic tissue in this study were higher compared to those of previous studies (Koopmans et al., 2006; Shen et al., 2012a, b; Liu et al., 2013), particularly the concentrations of 5-HIAA. Therefore, the 5-HIAA:5-HT is also higher when compared to that of similar studies. This difference could be explained by differences in the permeability of the blood–brain barrier in older animals when compared to younger animals. In a study conducted in rabbits, it was found that Trp uptake by the brain was decreased by 4-fold in the first 4 wk postnatally (Cornford et al., 1982). Similarly, the transport of Trp across the blood–brain barrier was significantly decreased in 12- and 24-month-old rats when compared to 2-month-old rats (Tang and Melethil, 1995). Furthermore, it is well understood that Trp competes with other large neutral amino acids (**LNAA**) which tend to be found in relatively higher quantities when compared to Trp (Pérez-Cruet et al., 1974; Pardridge, 1998; Shen et al., 2012a), indicating that both the timing and amount of supplemental dietary Trp could have an effect on its transport across the blood brain barrier. In this study, supplemental Trp was provided during the suckling period, whereas the aforementioned studies included additional Trp supplementation during the nursery period. Therefore, the results of the present study could be attributed to increased permeability of the blood–brain barrier in the suckling period, thus allowing for greater availability of orally supplemented Trp to the brain for 5-HT and 5-HIAA generation.

The results of the present study showed an inconsistent effect of oral Trp supplementation during the suckling period on the growth performance of pigs during the nursery period. An improvement in ADG and G:F was observed in week 4 postweaning, in pigs supplemented with oral Trp during the suckling period compared to pigs not supplemented with oral Trp; however, there was a decrease in G:F in week 2 postweaning, in the pigs supplemented with oral Trp, when compared to the pigs not supplemented with oral Trp. Previous research indicates that ghrelin, a key hormone involved in hunger and satiety cues, may influence the central serotonergic system, with ghrelin being shown to decrease serotonergic release from hippocampal slices (Ghersi et al., 2011) and hypothalamic synaptosomes (Brunetti et al., 2002), and altering the firing rate of cells in the dorsal raphe in vitro (Hansson et al., 2011), in rats. The complex relationship between Trp, ghrelin, and the central serotonergic system could explain why oral supplementation of Trp increased hypothalamic 5-HT and 5-HIAA, but consistent improvements to feed intake and growth performance were not observed throughout the entire nursery period, in the present study. These results are further supported by a similar study that found that although dietary Trp (5 g/kg of feed) increased serotonergic activity and successfully reduced salivary cortisol during social mixing stress; however, ADFI and behavior during mixing were not affected by the inclusion of dietary Trp in weaned pigs (Koopmans et al., 2006). Similarly, Shen et al. (2012b) and Koopmans et al. (2012) also found no effect of Trp supplementation on the eating behavior and ADFI of pigs. In contrast, Shen et al. (2015) evaluated the effects of short-term dietary supplementation of Trp in diets with a simultaneous reduction of LNAA found that supplementation of 0.8% Trp improved growth performance and was associated with the reduction of stress hormone concentrations of pigs during the social mixing period. Some positive effects of stress reduction could be seen in the present study, with pigs that received oral Trp during the suckling period exhibiting a tendency to visit the feeder more frequently in the 24 h immediately postweaning than pigs that did not receive Trp during the suckling period. It is worth noting that in this study, the space allowance per pig (0.72 m^2^) was greater than that of the minimum space allowance for pigs of this size (Wolter et al., 2000; Gonyou et al., 2006), and only 2 pigs were within each pen. Therefore, it would be reasonable to assume that in a commercial setting with a decreased space allowance per pig and a greater number of pigs per pen, the benefits of oral Trp supplementation during the suckling period on stress postweaning may be increased.

Studies have demonstrated that exposure to psychological stressors disrupts the population profile of intestinal microbiota (Galley et al., 2014). It is also known that there are interactions between microbes, enterochromaffin cells (**ECC**), and central nervous system (**CNS**) (Martin et al., 2018), and that 5-HT is produced by ECC present in the gastrointestinal tract. The gastrointestinal tract houses 95% of the body’s 5-HT in ECC and enteric neurons, whereas only 5% is stored in the CNS (Kim and Camilleri, 2000). Gastrointestinal 5-HT has a central role in the regulation of gastrointestinal motility and secretion, besides a selective pressure on the gut microorganisms to act on the serotonergic system to modulate their environment (Martin et al., 2018). Considering that Trp is a key molecule in the brain-gut-microbiome (**BGM**) axis producing serotonin and other metabolites involved in the neuroendocrine signaling (Ruddick et al., 2006), long term Trp supplementation can alter a pig’s intestinal microbiota (Liang et al., 2018), and understanding that the microbiota in turn can modulate Trp degradation via the kynurenine pathway (Martin et al., 2018), the effect of Trp on feed intake, growth performance and behavior probably is not just restricted to the 5-HT in the brain. Therefore, the interactions between the BGM axis and dietary Trp warrant further investigation to understand the mechanisms by which the BGM axis is related to the regulation of the stress response.

In conclusion, oral Trp supplementation to suckling piglets, from 14 to 20 d of age, increased hypothalamic concentration of 5-HT and 5-HIAA, potentially due to increased transport of Trp across the blood brain barrier in pigs of younger ages, which tended to increase the frequency of visits to the feeders after weaning, but had inconsistent effects on growth performance in the postweaning period. This study suggests that creep feeds with supplemental Trp for suckling piglets could be beneficial in enhancing the production of cerebral 5-HT and 5-HIAA, thereby influencing eating behavior and feed efficiency in the postweaning period, when pigs are under social mixing stress.

## Abbreviations

5-HIAA: 5-hydroxyindolacetic acid
5-HT: 5-hydroxytryptamine
ADFI: average daily feed intake
ADG: average daily gain
BW: body weight
CNS: central nervous system
ECC: enterochromaffin cells
G:F: gain to feed ratio
ME: metabolizable energy
